# Pre-residency publication and its association with paediatric residency match outcome—a retrospective analysis of a national database

**DOI:** 10.1007/s40037-017-0383-8

**Published:** 2017-11-13

**Authors:** Ronish Gupta, Mark L. Norris, Nicholas Barrowman, Hilary Writer

**Affiliations:** 1 Department of Pediatrics, Children’s Hospital of Eastern Ontario, Ottawa, Ontario Canada; 2 Clinical Research Unit, Children’s Hospital of Eastern Ontario Research Institute, Ottawa, Ontario Canada

**Keywords:** Internship and residency, Publishing, Curriculum

## Abstract

**Introduction:**

Scholarly activity is considered valuable in the resident selection process by candidates and program directors alike, despite existing literature suggesting applicants with scholarly work do not perform better in the match. These studies, however, are limited in that they have only measured whether candidates have successfully matched or not. To try and reconcile the existing disconnect in the value of pre-residency scholarly activity, we sought to deepen the understanding by investigating whether pre-residency publication is associated with a higher rank-order list match achievement.

**Methods:**

Anonymized data were collected from the Canadian Residency Matching Service for individuals matched to paediatric programs from 2007–2012. The primary analysis was to identify whether documentation of ≥1 pre-residency publication was associated with achieving a first-choice match. Secondary analyses included evaluating for an association between multiple pre-residency publications, academic presentations or a graduate degree and match outcome.

**Results:**

Of a total of 843 matched individuals, 406 (48.2%) listed ≥1 pre-residency publication and 494 (58.6%) matched to their first-choice program. The possession of ≥1 pre-residency publications was not associated with matching to a candidate’s first-choice program (odds ratio = 0.94 [95% confidence interval = 0.71–1.24], *p* = 0.66). Similarly, listing ≥2 publications, ≥3 publications, a graduate degree, or an academic presentation was not associated with achieving a first-choice match.

**Conclusions:**

The results provide increased support for the notion that in aggregate, candidate scholarly activity does not influence match outcome. Accordingly, it is recommended that medical student research activities are fostered with the goal to improve their skills as scientists, and not simply to achieve a better residency match outcome.

## What this paper adds

We aimed to build on the existing literature that suggests residency candidates with evidence of scholarly activity do not perform better in the match process. Studies to date have been limited to measuring whether a candidate successfully matched or not. We have deepened the analysis by determining that scholarly activity is also not associated with final position achieved on the candidate rank-order list, providing further support for existing match outcome literature. These results highlight the need to guide students participating in pre-residency research activities to do so to become better scientists, and not simply to perform better in the resident match process.

## Introduction

The process of resident selection typically involves reviewing prospective candidate files which include standardized examination scores, medical school grades, letters of reference, and scholarly activity, as well as an interview. Surveys of program directors across specialties, as well as data from the National Residency Matching Program (NRMP) in the United States, suggest that many of these characteristics contribute to a candidate’s overall ranking [1–6]. In particular, candidate scholarly activity is consistently assigned at least medium importance by programs in these studies.

Despite the stated value of candidate scholarly activity, published literature drawn from various specialties indicates that applicants who demonstrate evidence of scholarship, such as participating in the authorship of peer-reviewed publications, do not consistently perform better in the match process [7–10]. Examination of data from the NRMP suggests that matched and unmatched candidates appear to be fairly similar with respect to number of research experiences [11]. These results reveal an apparent disconnect between the way the resident selection process is understood to work, and the way it is actually working. This information is particularly important when considering that candidates go to great lengths to demonstrate evidence of scholarly activity for the purposes of matching a residency position. This may be inferred from the increasing numbers of medical students taking research years, with the most common reason being to ‘increase competitiveness for residency application’ [12]. In addition, significant rates of publication misrepresentation exist on applications across specialties [13–16], behaviour that has also caught the attention of the media [17].

The existing literature may, however, be missing an important outcome in the match process. All studies to date have been limited to comparing whether candidates ultimately did or did not match, without any inquiry of which position on their rank-order list they achieved. Although overall match is an important outcome, the reality is that for Canadian medical students in 2016, 94.5% of all applicants matched to a residency program in the first round of applications [18]. Understanding that securing a residency match is certainly a key outcome, with such high overall match rates, the candidate’s final rank-choice achieved may represent a more important and potentially modifiable target for applicants. It is assumed that a candidate’s preference would be to match to a program high on their rank-order list, as this preference in many cases has greater potential (or at least perceived) implications on factors related to their professional and personal life. For this reason, residency applicants will often feel it necessary to partake in activities such as research with the goal of helping to bolster their chances of a match that is high on their rank-order list [12].

To determine if a relevant outcome measure was being overlooked, we aimed to study the potential impact candidate scholarly work may have on residency match outcome by analyzing the associated position achieved on the candidate rank-order list. Specifically, we sought to identify whether individuals with journal publications listed on their residency applications were more likely to match to their first-choice program.

## Methods

This study was a retrospective analysis of a comprehensive centralized national residency match database.

### Study population

All Canadian medical graduates and international medical graduates who matched to a Canadian paediatric residency program in the first iteration were included. Candidates who went unmatched in the first iteration and matched to a Canadian paediatric program in the second iteration were also included, but analyzed as a separate outcome group. Individuals who may have submitted applications to paediatric programs but subsequently matched to a different specialty were not included. This was done to avoid confounding analysis of the paediatric application process with that of other specialties. Paediatrics was selected based on the authors’ familiarity with the discipline, but could also be considered a representative model due to it being a medium-sized field with both academic and community interests.

### Data collection

All applications to Canadian residency programs are managed by a centralized independent organization, the Canadian Residency Matching Service (CaRMS). Prospective candidates register with CaRMS and apply to desired residency programs after building their online profile, including research history (publications, presentations, and graduate degrees), academic transcripts, examination results, reference letters, medical student performance records, and personal essays. Following the interview period, candidates compile a rank-ordered list of their program preference (e. g. first-choice program, second-choice program, etc.), which is compared with the rank-ordered lists of candidates made by the residency programs. Finally, all candidates and programs undergo a match process employing a computerized algorithm. The algorithm uses candidates’ overall application scores with each program, and assigns them the highest possible program match from their rank-ordered list. It is possible for candidates to go ‘unmatched’ if the algorithm is unable to assign them a position. This occurs when the quotas of the programs they have applied to have already been filled with higher ranked candidates.

Anonymized records were collected retrospectively from the CaRMS database for all individuals who matched to a Canadian paediatric residency program in the first or second iteration from 2007 to 2012. Each record contained the number of peer-reviewed publications, number of academic presentations, and any graduate degrees listed in the applicant file. The primary outcome was whether a candidate was matched to their first-choice program or not. Secondary outcomes were overall candidate rank choice achieved, and whether a candidate was matched or unmatched in the first iteration. The decision not to collect additional demographic information (e. g. candidate age, gender) was made to avoid any risk of violating the confidentiality agreement of maintaining anonymity from source at the outset of the study.

### Sample size

Recent work indicates that 30% of paediatric residents in Canada have pre-residency publications listed in PubMed [19]. Data available from CaRMS suggest that approximately 60% of Canadian applicants will match to their first-choice program on their rank-order list [18]. The consensus of the research group was that a 20% difference between groups (0 vs. ≥1 publication) in the proportion of candidates matched to their first-choice program would be important. Fixing the probability of type-I error at 5%, a sample size of at least 230 would be needed in order to have at least 80% power to detect a 20% difference between groups. This would require approximately two years of CaRMS matching data. Given the desire to examine possible time trends, we collected six years of data, corresponding to the years 2007–2012.

### Statistical analyses

The CaRMS data detailing match rank outcome, number of publications, holding a graduate degree, and prior academic presentations were summarized descriptively, overall and by year. In order to identify any time trends, the data were analyzed by logistic regression vs. year as the predictor. The primary analysis was to evaluate the association between the primary outcome (achieving a first-choice program match) and whether the candidate had at least one publication listed on their residency application, using a Pearson’s chi-square test and logistic regression.

Secondary analyses included investigating the association between the primary outcome and other measures of scholarly work, namely graduate degrees, academic presentations, and authorship of multiple publications. We also investigated the association between measures of scholarly activity and achieving a first- or second-choice; or first-, second-, or third-choice program match. In addition, measures of scholarly activity were compared between candidates who matched in the first iteration and those who matched in the second iteration. Pearson’s chi-square tests and logistic regression were used in each of these cases.

We anticipated that peer-reviewed publications might have a different association with match outcome among candidates depending on whether they possessed a graduate degree. For this reason, a separate analysis of publications and match outcome was performed on subgroups of candidates separated by whether or not they held a graduate degree. Finally, a Spearman’s rank correlation was also used to measure the degree of association between candidate scholarly activity (number of publications or academic presentations) and match outcome considered as ranked variables. All statistical analyses were performed using R software version 3.1.0 [20].

### Permissions

The study protocol was approved by the research ethics board at the Children’s Hospital of Eastern Ontario. The protocol was also reviewed and data release approved by CaRMS.

## Results

**Fig. 1:**
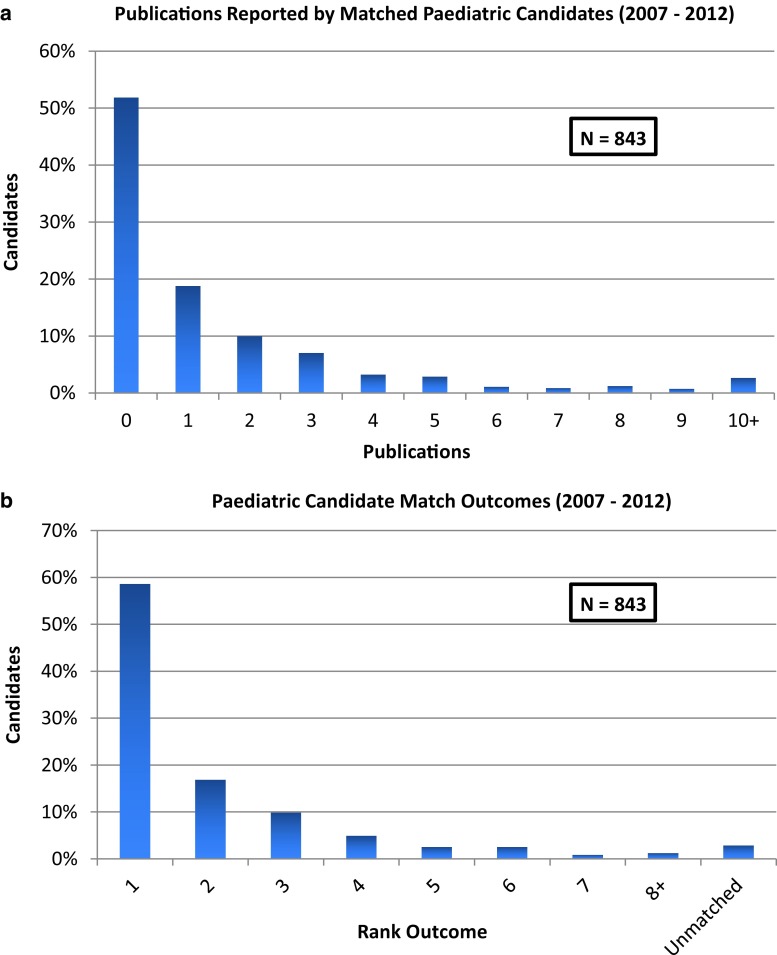
**a** Summary of publications reported on the residency applications by candidates matching to Canadian Paediatrics programs from 2007–2012. **b** Summary of match outcomes of candidates matched to Canadian Paediatrics programs from 2007–2012

A total of 843 individuals matched to paediatric residency programs in Canada from 2007–2012. The overall candidate publications and match outcome data are shown in Fig. 1. In total, 406 individuals (48.2%) had at least one publication listed on their application file, and 494 individuals (58.6%) matched to their first-choice program on their rank-order list. A summary of the other measures of candidate scholarly activity and match outcome data is outlined in Table 1. There were no statistically significant trends or differences in rates of scholarly activity or match outcome between years during the study period.

**Table 1:** Summary of publication and match data

Year	Number of candidates (%)						
	Total	≥1 Publications (%)	Graduate degree (%)	≥1 Academic presentation (%)	1^st^ choice match (%)	Top 3 choice match (%)	Unmatched^a^ (%)
2007	116	66 (57)	18 (16)	116 (100)	60 (52)	98 (84)	5 (4)
2008	125	56 (45)	17 (14)	125 (100)	74 (59)	107 (86)	5 (4)
2009	137	58 (42)	24 (18)	137 (100)	79 (58)	113 (82)	3 (2)
2010	149	64 (43)	20 (13)	149 (100)	80 (54)	121 (81)	8 (5)
2011	157	69 (44)	18 (11)	157 (100)	98 (62)	139 (89)	0 (0)
2012	159	93 (58)	26 (16)	159 (100)	103 (65)	141 (88)	3 (2)
Total	843	406 (48.2)	123 (14.6)	843 (100)	494 (58.6)	719 (85.3)	24 (2.8)

Baseline scholarly activity characteristics and match outcome results for all candidates matched to Canadian Pediatric Residency Programs from 2007–2012. There are no statistical differences between years of study

^a^Unmatched indicates candidates who were unmatched in the first iteration, and matched to a pediatric program in the second iteration.

Evaluation of the primary analysis revealed that there was no association between candidates having at least one publication listed on their applicant file and matching to their first-choice program (*OR* = 0.94 [95% CI = 0.71–1.24], *p* = 0.66). The detailed regression results of the primary analysis are shown in Table 2. In general, there were no significant associations between the various measures of candidate scholarly activity and their match outcome (Table 3). Academic presentations were not included in the final analysis, as all candidates had listed at least one presentation.

**Table 2:** Logistic regression results of primary analysis (≥1 publication as a predictor for 1^st^ choice match outcome)

Variable	β	Standard error	*p*-value	Odds ratio	95% CI
Intercept	0.45	0.10	–	–	–
≥1 Publication	−0.06	0.14	0.66	0.94	0.71, 1.24

*CI* confidence interval

**Table 3:** Relationships between candidate publications and match outcomes

	Matched program position on candidate’s rank list		
Scholarly activity	1^st^ choice	1^st^ or 2^nd^ choice	1^st^, 2^nd^, or 3^rd^ choice
≥1 Publication	0.94 [0.71–1.24]	0.89 [0.64–1.23]	0.69 [0.45–1.06]
≥2 Publications	1.15 [0.84–1.57]	0.84 [0.59–1.20]	0.71 [0.46–1.10]
≥3 Publications	1.23 [0.86–1.75]	0.81 [0.55–1.21]	0.63 [0.39–1.01]
Graduate degree	1.26 [0.84–1.90]	1.69 [0.99–2.88]	1.25 [0.66–2.37]

Relationship between various measures of candidate scholarly activity and match outcome for all individuals matched to paediatric programs in the first iteration from 2007–2012. Results shown are: odds ratio [95% confidence interval]

Table 4 shows the number of publications versus candidate match outcome achieved. When analyzed as ranked variables, there was no significant association between number of publications (Spearman’s rho = 0.007) or academic presentations (Spearman’s rho = 0.04) and position on rank-order list achieved.

**Table 4:** Candidate publication number separated by match outcome. Overall candidate rank choice achieved

	Overall candidate rank choice achieved					
Publications	1	2	3	4	5	6+
0	260	75	47	16	13	15
1	83	36	13	9	3	8
2	47	14	9	3	2	4
3	38	3	6	5	2	4
4	11	2	4	4	1	4
5	16	2	2	1	0	3
6	6	2	1	0	0	0
7+	33	8	1	3	0	0

Publications: Candidate first iteration match outcome separated by number of publications listed on residency application (individuals unmatched in the first iteration not shown)

Of the 819 candidates who matched in the first iteration, 702 (85.7%) had not listed a graduate degree. When candidates without a graduate degree were examined separately, there was no association between having listed at least one publication and matching to their first-choice program (*OR* = 1.10 [95% CI = 0.81-1.49], *p* = 0.61). Similarly, no association was found for the group of 117 candidates (14.3%) with graduate degrees (*OR* = 0.78 [95% CI = 0.31–1.99],* p* = 0.78).

Of the 843 total candidates matched to paediatrics programs during the study period, 819 (97.2%) matched in the first iteration. Twenty-four (2.8%) were unmatched in the first iteration and matched to a paediatric program in the second iteration. There were no differences between candidates who matched in the first or second iteration with respect to number of publications listed (*p* = 0.70), or those who held graduate degrees (*p* = 0.24).

## Discussion

Program directors and medical students both consider candidate scholarly activity to be an important factor in resident selection. Existing literature suggests, however, this is not the case. We aimed to reconcile this apparent disconnect by analyzing a novel, alternate match outcome, namely the position achieved on a candidate’s rank-order list. To date, studies have been limited to comparing successfully matched candidates to their unmatched counterparts. To our knowledge, this is the first study to analyze how candidates who have successfully matched to a particular specialty compare with each other, from the perspective of pre-residency scholarly activity.

Our results indicate that measures of candidate scholarly activity, including publications, graduate degrees, and academic presentations, are not associated with match outcome in terms of position on the rank-ordered list achieved. This is consistent with the existing literature demonstrating no differences in candidates who were matched vs. unmatched. Accordingly, our results do not provide credibility to the notion that candidate scholarly activity improves match outcome.

The results call into question numerous surveys of residency program directors that report peer-reviewed publications are assigned at least medium, if not high, importance when evaluating candidates [1–4]. Similarly, residency applicants believe scholarly activity to be an important part of their candidate file [3, 12], possibly more so than program directors do. This expectation also appears to be motivating increasing numbers of students to take research years during medical school [12]. It is also likely underlying the significant rates of publication misrepresentation found in residency applications across various specialties [13–16].

One potential reason for the discrepancies between this literature and our findings is that much of the available information from program directors and medical students is based on subjective questionnaires. In contrast, our results support the more objective outcome-based body of literature, and may be a more reliable representation of the overall selection process. Another possible reason for the discrepancy is that candidate standardized examination scores are emphasized in residency applications in the United States [21], where the majority of these studies have been conducted. In Canada, standardized examination scores carry less weight. An additional consideration is that candidates with scholarly work may preferentially apply and match to programs seeking applicants with scholarly work, and vice versa. Finally, it is possible that candidates who demonstrate scholarly activity are more likely to have received interview offers. In this particular case, our study would suggest that the benefit of scholarly activity may be limited to the point of obtaining an interview offer, and then confers no further advantage in the match process.

Although our findings have implications for applicants and educators, they are not entirely surprising. Candidate scholarly activity is but one element of a complex, multi-factorial selection process. Beyond resident selection, the importance of pre-residency publication also remains questionable. There is evidence to suggest that individuals with pre-residency publications are more likely to publish as residents [19, 22], and pursue academic careers [23, 24]. On the other hand, studies have also shown that pre-residency scholarly activity is not associated with subsequent evaluations of overall resident performance [25–28]. For these reasons, individual programs likely weigh the value of pre-residency publication in a highly variable fashion; largely dependent on the degree to which they value training academic physicians. It is worth considering that applicants’ awareness of a program’s value of scholarship, or lack thereof, may create an application bias.

As the amount of resources dedicated to medical student research increases, along with the pressure for them to produce scholarly work, it is prudent to ensure that their efforts are not motivated solely by the prospect of increased match success. This perspective may be gained informally from peers, but has also been emphasized in online discussion fora [29], and by professional residency matching companies [30]. Undergraduate medical educators may wish to become better aware of this hidden curriculum in their institutions. As an alternative goal, research and scholarly curricula for medical students should focus on training physicians capable of performing high-quality analyses of and/or contributions to the scientific literature. For selection committees, when evaluating candidates with scholarly activity, it may be valuable to explore why the students chose to pursue the activity and what they have learned, in addition to the final products. Finally, candidates without pre-residency scholarly activity may be reassured that they maintain competitive prospects during the process of resident selection.

### Limitations

Our results are based on an analysis of a large database. Information from individual records could not be verified; however, a quality assurance process was performed by CaRMS prior to data release. The percentage of candidates with publications (48.2%) was higher than that previously shown [19], and used for sample size determination (30%). The value of 30% was likely an underestimate as it was derived from a study limited to the PubMed database, and would not have captured candidate publications in peer-reviewed journals external to PubMed. Additionally, given the existence of publication misrepresentation on applications, the value of 48.2% may also partly be an overestimate of publication rate. The available database contained Canadian and international medical graduates records combined. Separation of the Canadian and international medical graduate groups may demonstrate different trends, however the international medical graduate group accounts for <10% of the total study population. The study excluded candidates who may have applied to paediatrics but matched to another specialty. This was done to avoid introducing confounders from the selection processes of other specialties, and from those who were interested in pursuing specialties outside of paediatrics. However, this may represent a source of selection bias in the group studied.

The study was also limited by the type and amount of information that was gathered from the CaRMS database. Collection of data such as candidate age, gender, and matched program identifiers would have permitted additional regression analyses using relevant covariates. However, this information was not obtained as part of individual records so as not to risk violating the ethics and confidentiality agreements of maintaining anonymity from source. This may have, for example, permitted the investigation of the possibility that candidates with scholarly work preferentially apply and match to programs seeking candidates with scholarly work, and vice versa. Publication features such as impact factor of the journal, candidate position on authorship list and publication timing (e. g. during medical school, graduate school) were also not collected in the present study, but represent interesting avenues for future work.
